# Editorial: Virtual surgical planning and 3d printing in head and neck tumor resection and reconstruction

**DOI:** 10.3389/fonc.2022.960545

**Published:** 2022-08-08

**Authors:** Yu-xiong Su, Florian M. Thieringer, Rui Fernandes, Sat Parmar

**Affiliations:** ^1^ Division of Oral and Maxillofacial Surgery, Faculty of Dentistry, The University of Hong Kong, Hong Kong, Hong Kong SAR, China; ^2^ Department of Oral and Maxillofacial Surgery, University Hospital of Basel, Basel, Switzerland; ^3^ Department of Oral and Maxillofacial Surgery, College of Medicine - Jacksonville, University of Florida, Jacksonville, FL, United States; ^4^ Department of Oral and Maxillofacial Surgery, University Hospitals Birmingham NHS Foundation Trust, Birmingham, United Kingdom

**Keywords:** computer-assisted surgery, virtual surgical planning, 3d printing, patient-specific implant, head and neck surgery, maxillofacial surgery, reconstruction, free flap

During the past two decades, we have witnessed rapid development of technology in the field of surgery. With the advancement of virtual surgical planning and medical three-dimensional (3D) printing (additive manufacturing), computer-assisted surgery (CAS) has revolutionized head and neck surgery and craniofacial surgery, leading to a new era of “digitalization and precision surgery” (1). CAS may refer to one or a combination of the following technologies: virtual surgical planning, navigation, 3D modelling, patient-specific surgical template/cutting guides, patient-specific implants, virtual reality (VR)/augmented reality (AR)/mixed reality (MR), and artificial intelligence (AI).

CAS consists of three phases (**Figure 1**). The first phase is the preoperative phase of virtual surgery and 3D-printing of patient-specific devices. While commercial services are currently available, in-house virtual surgical planning and 3D printing at the point-of-care have become increasingly popular in privileged centers (2, 3). Meanwhile, open-source software provides an alternative in low-resource settings (Ritschl et al.). The second phase is the intraoperative phase of precision-enhanced real surgery using patient-specific devices, navigation, and/or VR/AR/MR. The third phase is the postoperative phase of accuracy analysis, which is optional but beneficial in providing feedback for the surgical execution of preoperative planning (4). Evidence on the benefits of using CAS for head and neck reconstruction has been accumulating. Earlier, publications usually focused on technical aspects, and case reports or series with a low level of evidence. Recently, an increasing number of studies have provided a higher level of evidence regarding clinical efficacy, surgical accuracy, clinical outcomes, and oncology safety. These studies have advanced scientific research on CAS. Recently in some hospitals, virtual surgical planning and 3D printing for jaw reconstruction have become routine clinical practices.

**Figure 1:**
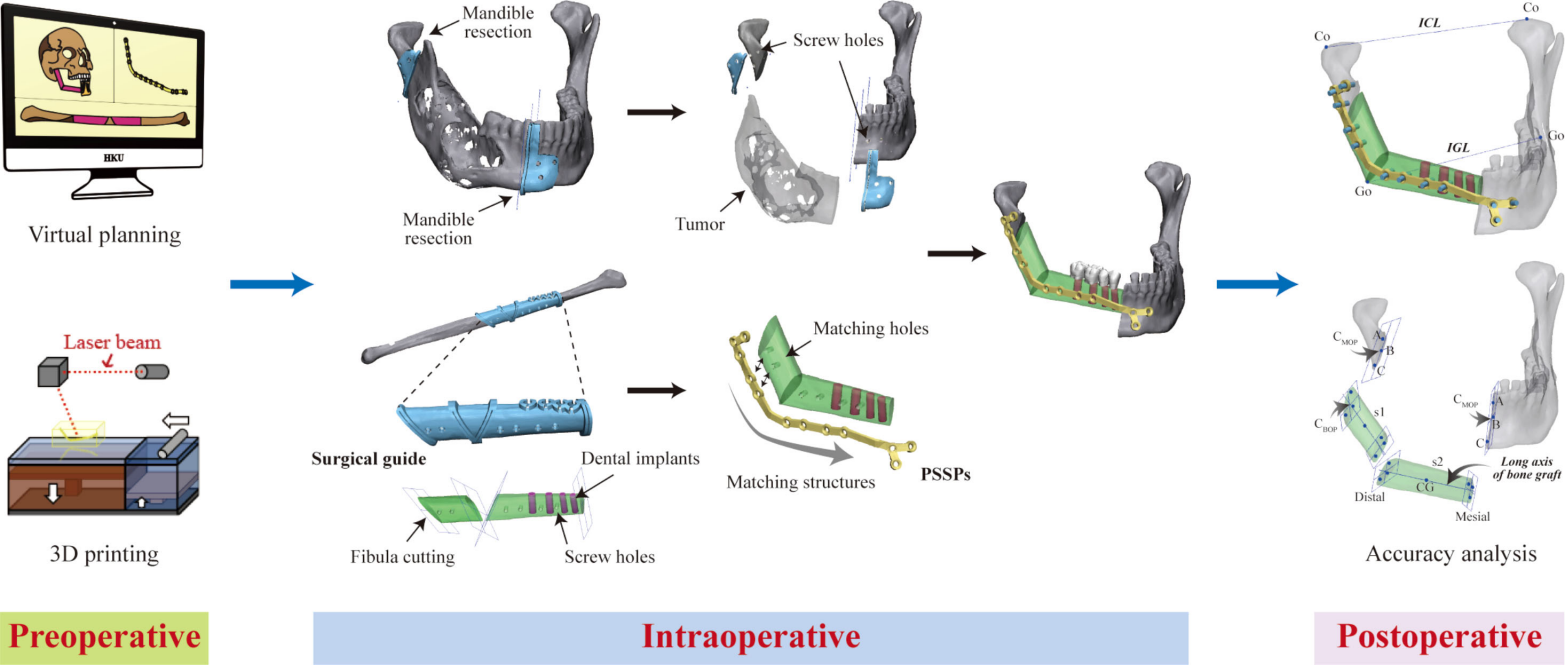
Schematic diagram of three phases of computer-assisted jaw reconstruction, including preoperative phase of computer panning and 3D printing, intraoperative phase of precision-enhanced surgery with the use of patient-specific devices, and postoperative phase of feedback of surgical execution by accuracy analysis.

The Research Topic of “virtual surgical planning and 3D printing in head and neck tumor resection and reconstruction” aims to collect the most recent evidence to popularize this cutting-edge technology in clinical practice. Altogether, 26 manuscripts from 185 contributing authors have been published on this Research Topic in Frontiers in Oncology and Frontiers in Surgery. We are excited to see that these publications cover the most advanced developments and reveal future research directions in this field.

## Combination of CAS with the new emerging technologies

Traditionally, CAS uses surgical guides to transfer virtual surgical planning into real surgery. The use of patient-specific surgical plates further enhances surgical accuracy and clinical outcomes. In recent years, an increasing number of researchers have combined the use of new emerging technologies, such as VR, AR, MR, navigation, AI, and tissue engineering, in computer-assisted head and neck reconstruction, showing that technological innovation is still the driving force in surgery. These new technologies have been used to assist tumor resection and reconstruction.

For tumor resection, AR with navigation is advantageous in delineating tumor extension, thus helping achieve negative surgical margins, especially in anatomical locations that are suboptimal for open access, such as the sinonasal, midface, and intraorbital (Taboni et al., Sahovaler et al., Yang et al., Garcia-Sevilla et al., Tang et al., Tel et al., Wu et al., Ha et al.). Tang et al. considered MR and surgical navigation had complementary advantages in tumor resection, thus the combination of both was recommended. These new technologies enhance the accuracy of tumor excision and increase oncological safety, while reducing the invasiveness of surgery.

For reconstruction, computed tomography (CT) angiography is widely used for perforator mapping in virtual surgical planning (Knitschke et al.).The AR-based protocol has been used to assist fibula flap skin paddle harvesting on a 3D-printed phantom (Cercenelli et al.). AI-enabled CT segmentation has also shown its advantages over manual segmentation during computer planning (5). Most recently, 3D printed bone scaffolds with tissue engineering have been used in jaw reconstruction, which highlights a promising minimally invasive approach in the future (6). A multidisciplinary research group in Basel used an engineered, vascularized, prefabricated bone graft for the reconstruction of maxillary defects. Although bone resorption was identified, this technique demonstrated the safety and feasibility of composite graft engineering for the repair of complicated head and neck defects (Ismail et al.).

## Accuracy and clinical outcomes of CAS

CAS not only enhances surgical efficiency but also improves clinical outcomes, including the accuracy of surgery (7, 8) Chen et al., Möllmann et al., Kang et al.). The studies in this Research Topic provide scientific data and evidence to support the benefits of the new technology, including precise tumor resection and satisfactory functional and aesthetic reconstruction outcomes.

Achieving surgical margin safety is of paramount importance for treatment outcomes. Previous studies have proved that predetermined resection margins during virtual planning do not compromise adequate surgical margin and oncologic outcome (9, 10). Wilkat et al. used navigation for mapping intraoperative frozen sections, leading to accurate 3D localization of the margins, which could be further incorporated into CT data for precise adjuvant radiotherapy planning. A higher rate of residual-free resections was reported in the CAS group than in the non-CAS group. Giannitto et al. used a 3D-printed specific tongue with *ex vivo* real-time magnetic resonance imaging to determine the orientation of the surgical margins. Regarding the accuracy of reconstruction, Pu et al. developed a lateral malleolus cap to overcome the sliding and rotational errors of fibula cutting guides and found that the accuracy of simultaneous dental implants could reach the accuracy of dental implants in native jaws. A preliminary analysis showed that the postoperative soft tissue contour of mid-face reconstruction was superior in the CAS group compared to that in the freehand group (Wang et al.). These studies provide solid evidence supporting the benefits of CAS in clinical practice.

## Learning curve and change of surgical plan

One of the major criticisms of CAS is its lack of flexibility during surgery. Although many concerns have been raised regarding the learning curve and changes in computer plans during surgery, few studies have focused on this important topic so far. We are delighted that this previously neglected issue has been addressed well in this Research Topic.

Zhu et al. used cumulative sum analysis to reveal a three-stage learning curve of CAS with the use of patient-specific implants, including the initial learning, plateau, and overlearning stages. Two independent studies performed in Europe and Asia investigated surgical adherence and unexpected changes in surgical plans (Ma et al., Pu et al.). With thoughtful preoperative planning and proper execution of surgery, surgeons can minimize the rate of unexpected change of surgical plans during surgery to as low as 5.6%, which has well addressed the criticism. Four clinical scenarios of unexpected changes were summarized, which could help junior surgeons prevent unfavorable situations and rationalize contingency strategies in CAS (Antúnez-Conde et al.). These studies provide valuable experiences for beginners to learn and follow, which can help shorten their learning curve.

## Dental rehabilitation and functional jaw reconstruction

With the use of virtual surgical planning and 3D printing in jaw reconstruction, we can achieve high surgical accuracy. This contributes to not only facial contour but also dental rehabilitation, which is important to increase the quality of life after oncological surgery. Virtual surgical planning and 3D printed patient-specific plates make simultaneous dental implantation during osseous free flap reconstruction easier and more controllable (Antúnez-Conde et al.). The design of “three-in-one” fibula surgical guide incorporates three major functions, including fibula segmentation, dental implantation, and positioning of patient-specific plates, which greatly facilitates the reconstructive surgery (11). Furthermore, with immediate loading, “Jaw-in-a-day” procedure can restore the teeth and function at the same time of tumor resection, especially for benign cases (12). In recent years, intraoperative navigation and AR have been incorporated into surgery to facilitate dental implantation in different bony flaps. The combination of AR with dynamic navigation reduced the deviation of dental implant in reconstructive patients with complex anatomical structure (Ochandiano et al.) Good implant success rates and optimal clinical outcomes of dental rehabilitation have been reported, which has laid the foundation for functional jaw reconstruction.

Alternatively, Korn et al. used a new design for patient-specific implant-borne dental rehabilitation in patients with large maxillary defects. This approach avoids osseous free flap surgery for jaw reconstruction, which shows that 3D printing technology may have other revolutionary potential to change the future of surgery.

## Summary

In summary, virtual surgical planning and medical 3D printing are transforming head and neck surgery by increasing predictability and repeatability, improving efficiency, enhancing resection and reconstruction accuracy, and facilitating dental rehabilitation and functional jaw reconstructions. With the incorporation of other emerging technologies, such as AR, MR, and AI, smarter and more intelligent surgery will become a reality in the near future.

## Author contributions

All authors listed have made a substantial, direct, and intellectual contribution to the work and approved it for publication.

## Acknowledgments

We would like to thank all the authors’ contribution to this Research Topic and eBook.

## Conflict of interest

The authors declare that the research was conducted in the absence of any commercial or financial relationships that could be construed as a potential conflict of interest.

## Publisher’s note

All claims expressed in this article are solely those of the authors and do not necessarily represent those of their affiliated organizations, or those of the publisher, the editors and the reviewers. Any product that may be evaluated in this article, or claim that may be made by its manufacturer, is not guaranteed or endorsed by the publisher.
